# Associations between Covariates and Pneumothorax Observations in CT-Guided Lung Biopsies

**DOI:** 10.3390/jcm11071958

**Published:** 2022-04-01

**Authors:** Nour Maalouf, Daniela Lavric, Lora Vasileva, Wolfram Lamadé, Jonas Apitzsch

**Affiliations:** 1 Department of Radiology and Nuclear Medicine, Helios Hospital Pforzheim, 75175 Pforzheim, Germany; daniela.lavric@helios-gesundheit.de (D.L.); lora.vasileva@helios-gesundheit.de (L.V.); jonas.apitzsch@helios-gesundheit.de (J.A.); 2 Department of General and Visceral Surgery, Helios Hospital Pforzheim, 75175 Pforzheim, Germany; wolfram.lamade@helios-gesundheit.de

**Keywords:** pneumothorax, chest CT, lung biopsy, biopsy needle

## Abstract

The purpose of this study is to assess the effect of nine covariates on the occurrence or absence of stable or symptomatic pneumothorax. Forty-three patients underwent CT-guided lung biopsies from January 2020 to January 2022 (24 m, 19 f, median age 70 years). All the interventions were carried out with a semi-automatic 18G needle and a 17G trocar in a prone or supine position. Different covariates were measured and correlated to the rate and severity of the pneumothoraces observed. Nominal two-sided *t*-test *p*-values for the continuous variables and Fisher’s exact test results for the categorical variables were conducted. The data included the lesion size, distance to the pleura, needle-pleura angle, age, gender, position during the procedure, and the presence of chronic obstructive pulmonary disease. Patients with an observed pneumothorax had an average angle between the needle and the pleura of 74.00° compared to 94.68° in patients with no pneumothorax (*p*-value = 0.028). A smaller angle measurement correlated with a higher risk of pneumothorax development. The needle-pleural angle plays a vital role in the outcome of a CT-guided lung biopsy. Correctly adjusting the needle-pleural angle can diminish the pneumothorax risk associated with a CT-guided lung biopsy. The study results show that as the needle’s angle deviates from the perpendicular, the pleural surface area experiencing trauma increases, and pneumothorax is more likely to occur.

## 1. Introduction

Patients benefit substantially from an image-guided lung biopsy in terms of therapy and treatment planning [1]. Lung biopsies are routinely needed for the correct diagnosis of various pulmonary nodules; however, they are not without complication risks, such as pneumothorax. Pneumothorax complications can be either major, involving hemothorax or requiring thoracostomy, or minor, neither resulting in shortness of breath nor needing chest tube insertion [2,3].

Nowadays, with the ongoing advancements in histopathological, immunohistochemical, and molecular analyses prior to treatment, there is an increased demand for tissue biopsies [4]. A recent meta-analysis by Heerink et al. of 12,753 computed tomography (CT)-guided transthoracic lung biopsy procedures reported a 25.3% pneumothorax incidence either during the procedure or immediately thereafter [5]. In a retrospective study conducted by Kuriyama et al. [6], it was found that 11.2% of the CT-guided lung biopsies performed were interrupted and discontinued by pneumothorax.

The papers reporting the aforementioned studies, and a couple of others, discuss lung biopsy factors that may lead to pneumothorax. This paper correlates additional covariates to the observed rate and severity of pneumothoraces. Additionally, a hypothesis for the pathologic mechanism is proposed to explain the findings.

## 2. Materials and Methods

During the period from January 2020 to January 2022, 43 patients (24 males, 19 females; median age 70 years; age range 51–86 years) underwent a computed tomography (CT)-guided biopsy of the lung. All the CT-guided lung biopsies were conducted with an 18GB semi-automated TruCut18G (Möller Medical GmbH, Fulda, Germany) and a 17G trocar in a supine or prone position (21 and 22 patients, respectively).

The indicator thresholds that were considered in this procedure were age; gender; position; the presence of chronic obstructive pulmonary disease (COPD); lesion size; distance to the pleura; and left, right, and minimum angles at which the needle passes through the pleura.

The presence of COPD was assessed according to each patient’s files and the presence of emphysema on a thoracic CT scan.

The post-procedural examination stratified the patients into three categories:

- Patients with no pneumothorax;
- Patients with stable pneumothorax;
- Patients with symptomatic pneumothorax.

The patients with a non-expanding and an asymptomatic pneumothorax were classified under “stable pneumothorax”, whereas patients who were experiencing shortness of breath, chest pain, and chest tightness were classified under “symptomatic pneumothorax”.

All the patients signed an informed-consent form more than 24 h prior to the intervention. Ethical approval was given by the local ethics committee (F-2021-038).

### 2.1. Inclusion and Exclusion Criteria

This observational study included 43 patients that underwent CT-guided lung biopsies and were classified to 3 groups: no pneumothorax observed, stable pneumothorax, and symptomatic pneumothorax requiring a chest tube.

Exclusion criteria were the following: a lesion diameter of <4 mm, INR of >1.5, and an incapacity to follow instructions or a refusal of the procedure.

### 2.2. Semi-Automated TruCut 18G Needle

The 18G biopsy needle (Möller Medical GmbH, Fulda, Germany) has a semi-automated Tru-Cut design. It is composed of a central sharp stylet surrounded by a hollow cylindrical sheath. The cylinder is 1 cm long, resulting in a 7.854 mm^3^ sample volume. The Tru-Cut design needle is a convenient and valuable tool, and its use has become a standard practice in diagnostic lung biopsies [1].

### 2.3. 17G Trocar

The 17G trocar (Möller Medical GmbH, Fulda, Germany) acts as a guide for the 18G needle and facilitates multiple biopsy entries. A 17G trocar, a gauge bigger than the 18G needle used in this study, was chosen to ensure optimal outcomes. By using a trocar, we could hamper excessive tissue trauma, which the biopsy could have caused.

### 2.4. Biopsy Protocol

In accordance with the previous imaging, a selective CT scan of the region of interest was obtained at end-expiration breath-hold. A 128-slice Siemens Somatom Definition Edge CT (Siemens, Forchheim, Germany) was used with the following protocol of a 20 mA current at 120 kV, and for 3 mm slice thickness.

After the intervention was planned and the puncture site was disinfected, local anesthesia (mepivacaine 1%) was administered, and a small skin incision was made. The coaxial needle was placed at the margin of the lesion. The 18G needle was introduced through the trocar and, with its rapid-firing mechanism, a biopsy was performed. The obtained samples were directly fixated in formaldehyde and were subjected to histopathological analysis. After the biopsy needles were removed and the puncture site was closed with a sterile patch, a low-dose non-contrast control scan of the chest was performed in end-expiration to assess the intervention-related complications.

There was never more than a single pleural passage of the trocar needle.

### 2.5. Measurement Yield

The angle was measured digitally while the needle was fully visible in the transverse plane, ensuring that the only variable angle was that in the mentioned plane. The right and left angles were measured with respect to the patient. The measurements were conducted using the picture archiving and communication system (PACS)—CHILI (CHILI GmbH, Dossenheim, Germany) system for angle measurements. This added to the consistency and reliability of the study results by eliminating the variability in the angle with respect to the coronal plane.

### 2.6. Statistical Analysis

Covariates related to the patient, target lesion, and the biopsy procedure were recorded (age; gender; COPD; lesion size; distance to the pleura; and left, right, and minimum angles) and evaluated in a multivariate analysis. The aforementioned data were recorded by the interventional radiologist and retrospectively evaluated with the help of the contingency tables below.

The data analysis was carried out through the binary classification of the patients into two categories according to the development of pneumothorax in order to eliminate any possible bias that could occur if the angles were categorized according to their ranges.

Four stratified analyses were conducted through pneumothorax observation: a nominal two-sided *t*-test for the continuous variables and Fisher’s exact test for the categorical variables. Statistical analysis was performed by Biomedical Statistical Consulting (1357 Garden Road, Wynnewood, PA 19096, USA).

## 3. Results

From January 2020 to January 2022, 43 patients underwent CT-guided lung biopsies, and different covariates were documented throughout this study. Table 1 shows the number of observations made, along with the calculated mean values obtained and the respective standard deviations of the nine different covariates used to assess the risk of pneumothorax development. Out of the 43 patients, 3 had their lesions directly adjacent to the thoracic wall, which decreased the number of subjects from 43 to 40 in looking at how the distance to the pleura (mm) acts as a risk factor.

**Figure 1:**
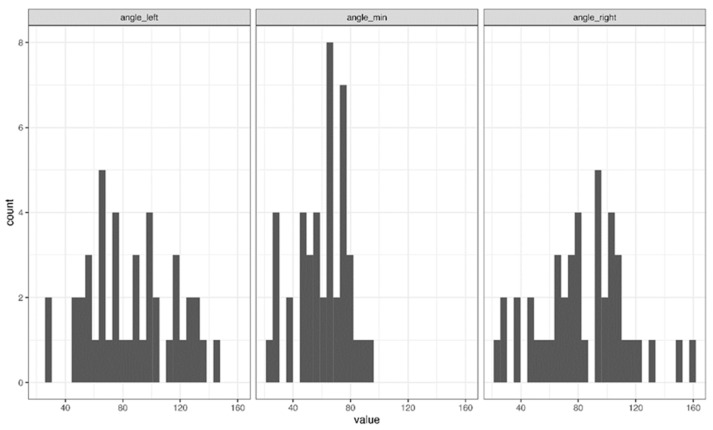
Histogram plot with various angles to the pleura.

Furthermore, the values of the needle-pleural angles (the left, right, and minimum angles) were displayed in three different histograms (Figure 1). The minimum angle was the lower of the right and left angle values; since the chest is curved, the sum of the right and left angles will not always be 180 degrees. The results of this observational study are shown in the following contingency tables.

As shown in Table 1, the patients had a mean age of 70.12 years (SD = 10.35). There were 24 males (56%) and 19 females (44%). Furthermore, 21 (48.8%) assumed a supine position, while 22 (51.2%) assumed a prone position, and 16 (37.2%) had COPD. The patients had a 33.95 mm (SD = 26.21) average lesion size and a 14.87 mm (SD = 14.92) average lesion distance to the pleura.

Table 2 presents the results of the first group of patients with observed pneumothorax. The right, left, and minimum angle measurements were in the range of 44.8–124.8°. The significance of the degree of the right angle is shown in Table 2 (*p* = 0.028). Table 2 shows that the binary pneumothorax variable was noted in 24 of the 43 cases in this study. A nominally statistically significant difference in the right angle was observed in the patients with pneumothoraces with smaller right angles: 74.00° (SD = 29.17°) versus 94.68° (SD = 30.15) (two-sided *t*-test *p*-value = 0.028). A similar directional observation was noted for the left angle.

Additionally, Table 3 shows a stratified analysis of the clinically stable pneumothorax. The patients with a clinically stable pneumothorax were observed to have a smaller left angle (*p* = 0.067); however, a statistically significant threshold was not quite reached.

The stratified analysis was repeated for the observations of the symptomatic pneumothorax. No statistically reliable associations were noted. The calculated minimum angle had no statistically significant effect on the occurrence of pneumothorax. None of the covariates displayed any statistically reliable correlation to symptomatic pneumothorax, as shown in Table 4. It is worth noting that the pneumothorax that occurred during the intervention when the needle was still inside the lung parenchyma was included in Table 2; however, the pneumothorax resolved spontaneously after removing the needle and was no longer detectable by the time of the control scan after the biopsy was over. Therefore, it was not included in Table 3 and Table 4.

**Figure 2:**
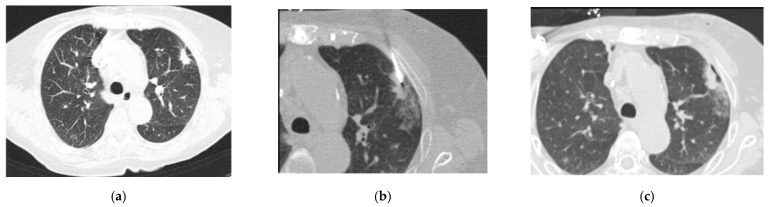
Axial CT images of the lungs conducted with the use of 90 mAs at 120 kV, and a 3 mm slice thickness with a 2 mm increment: (**a**) a pulmonary mass seen on the left, immediately before the biopsy; (**b**) the needle entering the pleura at an angle away from the perpendicular; and (**c**) a stable pneumothorax observed in the post-biopsy CT scan.

Figure 2a–c shows a pulmonary mass that was punctured during our study. Due to the adjacent rib, the needle’s angle to the pleura had to be rather shallow, as can be seen in Figure 2. The post-interventional control scan (Figure 2c) shows a small, clinically stable pneumothorax in the access pathway.

**Figure 3:**
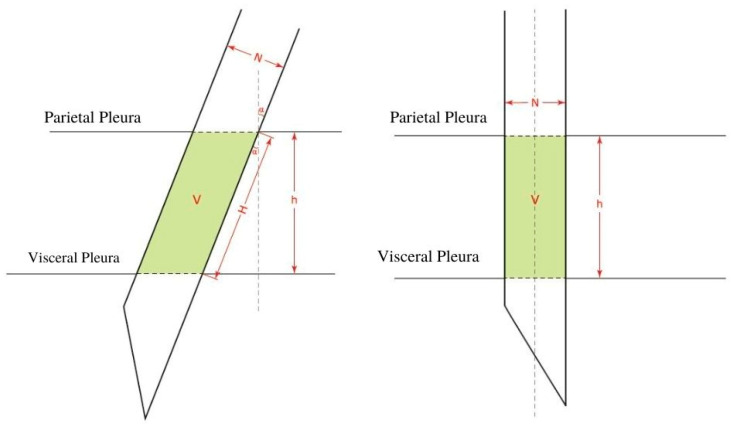
The volume occupied by the needle at an angle α (**left**), and the volume occupied by the needle as the needle hits the parietal pleura at a 90° angle (**right**). V: the volume occupied by the needle. H: the line formed by the needle going at an angle between the parietal pleura and the visceral pleura. h: the straight line between the parietal pleura and the visceral pleura. N: the diameter of the needle. α: the angle between the needle and a perpendicular line to the pleural membrane.

**Theorem** **1.** *We hypothesize that as the needle’s angle deviates from the perpendicular and as the path that the needle has to travel through within the pleural cavity, and consequently, the space that it will take up therein increase, the pleural surface area experiencing trauma likewise increases (Figure 3). This explains why as the angle deviates from the perpendicular, the affected area increases (the cosine of the angle decreases). In addition, if a larger needle is used, the area will increase as a function of the square of its radius, and thus, will cause significantly more trauma.*

As the needle punctures the tissue at an angle deviating from the perpendicular, the puncture site resembles an ellipse rather than a circle.

Let “N” represent the diameter of the needle; this diameter is constant and represents the minor diameter of the ellipse.

Let “Y” represent the major diameter, which changes according to the angle.

The area of an ellipse is π multiplied by the minor radius (N/2) multiplied by the major radius (Y/2).

We know that

A_Ellipse_ = π × (N/2) × (Y/2)

and

Y = N/cos α;

then,

A_Ellipse_ = π × (N/2) × (N/2cos α),

as well as

A_Ellipse_ = π/4 × (N^2^/cos α).

By definition, the volume (V) of a cylinder is the product of its base and height (H):

V_Cylinder_ = π × r^2^ × H

and

cos α = h/H;

so,

H = h/cos α

by substitution,

V_Ellipse_ = π × r^2^ × h/cos α.

V_Ellipse_ shows the relationship between the area and volume of the tissue, which is affected by the size and angle (α) of the needle.

## 4. Discussion

There was one variable that was found to have a statistically significant correlation with the rate and severity of pneumothoraces in this study: the needle-pleural angle. The analysis of the lung biopsies performed in our study showed that the smaller the angle is, the higher the risk of developing pneumothorax. We observed that the patients who underwent a lung biopsy with an acute needle-pleural angle almost perpendicular to the pleura had a lower risk of developing pneumothorax. This is in coherence with the recent study by Sheikh et al. who assessed 208 patients with 215 lung–mediastinal lesions after a CT-guided lung biopsy, and demonstrated a significant correlation between the needle-pleural angle and the presence of pneumothorax (*p* = 0.0200). Above all, the needle-pleural angles between 80 and 90 degrees had the lowest rate of pneumothorax at 14.8% [7]. Conversely, the patients who had a smaller needle-pleural angle (deviating away from the perpendicular) presented a high tendency to develop pneumothorax. The 24 patients with an observed pneumothorax had an average needle-pleural angle of 74°, and the patients with no observed pneumothorax had an average needle-pleural angle of 94.68°. Of the 24 patients with pneumothorax, 5 had an inapparent stable pneumothorax, where the average left needle-pleural angle was 61.80°. These results are in accordance with the findings of Ko et al. [8] and Saji et al. [9], who showed that needle-pleural angles of less than 80° have a higher risk of developing pneumothorax. The results obtained are also consistent with the recommendation provided by Birchard [10] to cross the pleura perpendicularly rather than obliquely. In a study conducted by Yeow et al. [11], the effect of the needle-pleural angle was eclipsed by the impact of the small lesion size. A possible explanation for this discrepancy in results, whereby we did not see the lesion size as statistically significant as shown by Yeow et al., is that they analyzed the lesion size parameter as interval data, while we analyzed as continuous numerical data. In an experiment conducted by Billich et al. [12], no correlation was found in a total of 70 patients between the lesion size and the presence of pneumothorax. In a recent retrospective analysis of 179 patients undergoing a lung biopsy, the mean distance from the skin to the lesion was not statistically different among patients with and without pneumothorax (*p* > 0.05) [13]. This finding is in agreement with our results (*p* = 0.179). Direct or angulated CT-guided needle placements were used in this study, and the double-angulated approach was never used. Busser et al. [14] demonstrated that the direction of the angulation influences and determines the difficulty of needle placement. The other variables that were observed in this study, such as age, gender, position, and the presence of COPD, had no effect on the pneumothorax occurrence or severity. The COPD patients in this study showed no significant difference from the non-COPD patients (*p* = 0.717). However, the relevant literature exposed contradictory results, for which reason further analysis is needed in the future. Contradictory results may be due to a smaller sample size as in this study, compared to a retrospective study performed by Heck et al. [15] in which an 85-patient sample showed a significant association between the occurrence of a pneumothorax requiring chest tube placement and the presence of COPD. Abnormal lung function was listed as a contraindication for a lung biopsy [16]. This finding was later reinforced by Boskovic et al. [17], who emphasized the role of COPD as a major risk factor for biopsy-related pneumothorax.

Of the ten patients needing chest tube placement in this study, five underwent a biopsy in the supine position. This can be explained by the fact that the anterior ribs move more than the posterior ribs, the anterior intercostal spaces are narrower than their posterior counterparts, and the prone position constricts the field of vision of the patient; thus, this decreases the patient’s anxiety and agitation [18]. According to Veltri et al. [19], when the patient has to be placed in the supine position, patient education becomes a priority to decrease the patient’s anxiety and movement. To protect the integrity of the pleura and lung parenchyma, the interventional radiologist performed a single pleural puncture coaxially. This prevented any unnecessary pleural damage and fissure [18]. Moreover, the coaxial technique seems more advantageous than the single-needle technique [20], and was shown to decrease the risk of pneumothorax development in a retrospective analysis of 485 patients conducted by Zhang et al. [21]. In a 2020 study, Huo et al. looked at 36 articles, including 23,104 patients, and presented the pooled incidence for pneumothorax at 25.9% and 6.9% for chest drain insertion [22]. The incidence of iatrogenic pneumothorax has often been reported and, according to Choi et al. [23], ranged from 8% to 61%, with 10.4–17.4% of the patients requiring chest tube placement.

Since many covariates play a role in the development of pneumothorax, it is apparent that decreasing its prevalence should be tackled on several levels. Even if a patient has an increased risk of developing pneumothorax, taking precautions can significantly lower the risk. The precautions can include an access route with an angle close to 90° and the use of a smaller needle. With respect to sample size and assessed risk, 18G is the preferred needle size for intrathoracic biopsy procedures; the use of a larger needle has a higher probability of pneumothorax development and chest tube placement [24]. The 18G needle is known to create minimal tissue trauma and organ deformities [25]. In addition, the clear-cut and swift mechanism of the semi-automated Tru-Cut needle yields an essential core tissue sample. Our study was limited to 43 patients and a single experienced operator (with 15 years of experience in interventional radiology). Other limitations of the study include the observational design and the inability to include external factors or experiment with new techniques. However, we believe that this study included all the variables needed to apprehend the risk factors of pneumothorax development and to decrease its occurrence during a CT-guided lung biopsy in clinical routine.

## 5. Conclusions

The needle-pleural angle plays a vital role in the outcome of a CT-guided lung biopsy. Adjusting the needle-pleural angle closer to 90° can diminish the risk of pneumothorax development associated with a CT-guided lung biopsy.

## Figures and Tables

**Table 1:** Descriptive statistics of the overall sample.

	*n*	Estimate
Lesion Size, Mean (SD)	43	33.95 (26.21)
Distance to Pleura, Mean (SD)	40	14.87 (14.92)
Angle Left, Mean (SD)	43	85.53 (30.04)
Angle Right, Mean (SD)	43	83.14 (31.04)
Angle Minimum, Mean (SD)	43	60.14 (17.55)
Age, Mean (SD)	42	70.12 (10.35)
Gender = Male, *n* (%)	43	24 (55.8)
Position = Supine, *n* (%)	43	21 (48.8)
COPD = Yes, *n* (%)	43	16 (37.2)

SD, standard deviation; COPD, chronic obstructive pulmonary disease.

**Table 2:** Stratification by the observed pneumothorax.

	Observed		Not Observed		
	*n*	Estimate	*n*	Estimate	*p*-Value *
Lesion Size, Mean (SD)	24	29.73 (19.63)	19	39.28 (32.50)	0.239
Distance to Pleura, Mean (SD)	22	17.76 (15.34)	18	11.33 (14.00)	0.179
Angle Left, Mean (SD)	24	92.54 (30.93)	19	76.68 (27.12)	0.086
Angle Right, Mean (SD)	24	74.00 (29.17)	19	94.68 (30.15)	0.028
Angle Minimum, Mean (SD)	24	57.17 (16.03)	19	63.89 (19.07)	0.216
Age, Mean (SD)	23 ^†^	69.00 (9.69)	19	71.47 (11.22)	0.448
Gender = Male, *n* (%)	24	14 (58.3)	19	10 (52.6)	0.948
Position = Supine, *n* (%)	24	11 (45.8)	19	10 (52.6)	0.892
COPD = Yes, *n* (%)	24	10 (41.7)	19	6 (31.6)	0.717

* Nominal two-sided *t*-test *p*-values for the continuous variables and Fisher’s exact test for the categorical variables. ^†^ One of the patients had two different procedures; thus, their age was not repeated.

**Table 3:** Stratification by the clinically stable pneumothorax.

	Observed		Not Observed		
	*n*	Estimate	*n*	Estimate	*p*-Value *
Lesion Size, Mean (SD)	5	23.32 (16.15)	37	36.06 (27.11)	0.314
Distance to Pleura, Mean (SD)	2	15.00 (8.49)	37	14.78 (15.46)	0.984
Angle Left, Mean (SD)	5	61.80 (11.69)	37	87.95 (30.49)	0.067
Angle Right, Mean (SD)	5	106.20 (5.89)	37	80.78 (31.92)	0.086
Angle Minimum, Mean (SD)	5	61.80 (11.69)	37	60.05 (18.52)	0.839
Age, Mean (SD)	4 ^†^	68.50 (6.24)	37	70.51 (10.79)	0.718
Gender = Male, *n* (%)	5	3 (60.0)	37	20 (54.1)	1.000
Position = Supine, *n* (%)	5	1 (20.0)	37	20 (54.1)	0.341
COPD = Yes, *n* (%)	5	2 (40.0)	37	13 (35.1)	1.000

* Nominal two-sided *t*-test *p*-values for the continuous variables and Fisher’s exact test for the categorical variables. ^†^ One of the patients had two different procedures; their age was not repeated.

**Table 4:** Stratification by the symptomatic pneumothorax.

	Observed		Not Observed		
	*n*	Estimate	*n*	Estimate	*p*-Value *
Lesion Size, Mean (SD)	10	25.38 (27.44)	33	36.55 (25.69)	0.242
Distance to Pleura, Mean (SD)	10	22.44 (15.82)	30	12.34 (13.98)	0.063
Angle Left, Mean (SD)	10	89.90 (23.59)	33	84.21 (31.94)	0.606
Angle Right, Mean (SD)	10	78.90 (23.74)	33	84.42 (33.15)	0.628
Angle Minimum, Mean (SD)	10	64.60 (10.51)	33	58.79 (19.11)	0.365
Age, Mean (SD)	10	67.40 (8.93)	32	70.97 (10.74)	0.348
Gender = Male, *n* (%)	10	6 (60.0)	33	18 (54.5)	1.000
Position = Supine, *n* (%)	10	5 (50.0)	33	16 (48.5)	1.000
COPD = Yes, *n* (%)	10	5 (50.0)	33	11 (33.3)	0.561

* Nominal two-sided *t*-test *p*-values for the continuous variables and Fisher’s exact test for the categorical variables.

## Data Availability

The data presented in this study are openly available in FigShare at [10.6084/m9.figshare.19491293]. 1. Maalouf, N.; Christoph Apitzsch, J. Table_Anonymized.xlsx2022.
